# Early onset first-episode psychosis during treatment with thalidomide for refractory ulcerative colitis: a case report

**DOI:** 10.1186/s13256-019-2106-8

**Published:** 2019-06-08

**Authors:** Maria Giuseppina Petruzzelli, Lucia Margari, Sara Ivagnes, Roberto Palumbi, Francesco Margari

**Affiliations:** 1 Child Neuropsychiatry Unit, Department of Basic Medical Sciences, Neuroscience and Sense Organs, University of Bari “Aldo Moro”, Policlinico Piazza, G. Cesare 11, 70124 Bari, Italy; 2 Psychiatry Unit, Department of Basic Medical Sciences, Neuroscience and Sense Organs, University of Bari “Aldo Moro”, Policlinico Piazza, G. Cesare 11, 70124 Bari, Italy

**Keywords:** Schizophrenia spectrum disorders, Neuroinflammation, Inflammatory bowel disease, Immunological dysregulation

## Abstract

**Background:**

Inflammatory bowel disease and schizophrenia spectrum disorders are complex and multifactorial conditions characterized by great variability of age at onset, clinical presentation, and longitudinal course. Several lines of evidence suggested different connections among immunological dysregulation, gastrointestinal inflammation, and psychosis, but to date many controversial issues still exist in this field.

**Case presentation:**

We present the case of a 14-year-old Caucasian boy with refractory ulcerative colitis, admitted to the Child Neuropsychiatry Unit of the Polyclinic Hospital of Bari in the course of his first-episode psychosis. He showed an acute onset of psychotic symptomatology during treatment with thalidomide, an immunomodulatory drug used in the experimental therapy of refractory inflammatory bowel disease. Thalidomide was discontinued and orally administered mesalazine was restarted. In addition, treatment with antipsychotics and mood stabilizers was introduced with gradual improvement of psychotic symptoms. According to *Diagnostic and Statistical Manual of Mental Disorders*, Fifth Edition criteria, a diagnosis of partial remission of a first episode of schizoaffective disorder was formulated after a 6-month follow-up. Throughout this period, both psychopharmacological and mesalazine-based gastrointestinal treatments were maintained with partial remission of psychiatric and gastrointestinal symptoms.

**Conclusions:**

We propose that refractory ulcerative colitis and psychosis might represent different manifestations of a common pathological pathway. However, it is also conceivable that thalidomide may have played a role in promoting the manifestation of psychotic symptoms in an individual with a specific vulnerability to schizoaffective disorders. Further investigations are needed to improve our knowledge regarding the complexity of brain–gut interactions, thus improving the management of co-existing inflammatory bowel and schizophrenia spectrum disorders.

## Introduction

Schizophrenia spectrum disorders are considered a heterogeneous group of conditions with a multifactorial etiopathogenesis. Current evidence suggests that the neurodevelopmental model best explains the pathogenesis of schizophrenia, and that the interaction of genetic background and multiple environmental risk factor exposures might cause disease pathogenesis. While early onset (before 13 years) appears to be quite rare, the rate of onset increases during adolescence, with a peak age ranging from 18 to 30 years [1]. Although neurobiologically and phenomenologically continuous with its adult counterpart, childhood-onset schizophrenia represents a more severe disorder, with more prominent pre-psychotic developmental disorders, brain abnormalities, and genetic risk factors [2].

In the last decades, multiple lines of evidence support the association between autoimmunity/inflammation and schizophrenia spectrum disorders [3, 4]. Overlaps in clinical course, in addition to epidemiological and genetic associations, raised the possibility that autoimmune mechanisms may underlie some psychoses, potentially offering novel therapeutic approaches [5]. In more recent years, the focus of the research regarding the relationship between autoimmune diseases and schizophrenia has been narrowed to gastrointestinal (GI) disorders [6]. In the context of the intricate interaction between the gut and the brain, the normal ecological balance of gut microorganisms plays an important role. Disorders in the composition and quantity of gut microorganisms, the so-called gut microbiota, have been reported to be associated with various central nervous system diseases [7] and current evidence suggests that probiotic supplementation could improve mild and moderate depressive symptoms [8]. On the other hand, a recently published systematic review found that there is a paucity of clinical studies to support the benefits of probiotic supplementation in patients with schizophrenia [9]. At the turn of the twenty-first century, inflammatory bowel disease (IBD) has become a global disease with accelerating incidence in newly industrialized countries with a prevalence surpassing 0.3% [10]. Ulcerative colitis (UC) is a form of IBD with diffuse inflammation of the rectal and colonic mucosa, manifesting with abdominal pain, diarrhea, bleeding, and weight loss. The pathogenesis of IBD, which is only partly understood, is thought to arise from dysregulation of the innate and adaptive immune systems, leading to an abnormal inflammatory response to commensal bacteria in genetically susceptible individuals [11]. The incidence rate of UC may vary from 0.5 to 31.5 per 100,000 people each year, depending on the studied population; the majority of patients with UC are in the age group of 30–40 years at diagnosis, while pediatric IBD, so defined when the age at onset is < 19 years, covers approximately 5–25% of patients [12]. UC displays a chronic clinical course, characterized by periods of exacerbation and remission, which may occur either spontaneously or in response to treatment [12]. UC rarely exists in isolation but is rather part of a complex matrix of disorders arising in patients over time [13]. Psychiatric comorbidity in IBD is well known, but to date the nature of the relationship between these two diseases has not been fully understood and is still a matter of debate. The most common psychiatric disorders in IBD are depression and anxiety, whereas data on other conditions, such as bipolar disorders and psychoses, are limited [14, 15].

In the current paper we present the case of an adolescent patient with a severe form of early onset UC, refractory to conventional therapies, manifesting an early onset first-episode psychosis (FEP) during treatment with thalidomide.

## Case presentation

In April 2018, a 14-year-old Caucasian boy with acute onset of affective FEP was referred to the Child Neuropsychiatry Unit of the Polyclinic Hospital of Bari. Since the age of 12 he presented with debilitating intestinal symptoms as mucohemorragic diarrhea (discharge frequency, > 10/day), tenesmus, and abdominal pain, resulting in severe disability impairing his general and social well-being. He was diagnosed as having UC on the basis of clinical, laboratory, instrumental, and histologic criteria. In accordance with the “Guidelines for Management of Pediatric Ulcerative Colitis” [16], he was treated with conventional therapies (mesalazine, prednisone, metronidazole, azathioprine, and biological agents such as infliximab and adalimumab) with no clinical response. Before elective surgery, a medical treatment with thalidomide was started, as an off-label option for patients with primary refractory IBD, and a clinical response was gradually observed. Two months later, he showed an acute onset of irritable mood, decreased need for sleeping, abnormally increased activity, disorganized behavior and speech, and thought alterations including inflated self-esteem and flight of insight-lacking ideas. These symptoms prompted the discontinuation of thalidomide and a mesalazine-based treatment was restarted. After admission at our Child Neuropsychiatry Unit, he was found to have no history of obstetric complications, neurological or psychiatric diseases, or substance abuse and no psychopathological symptoms prior to this acute episode. His parents reported a normal achievement of early childhood neurodevelopmental milestones and a normal intelligence quotient (IQ) was assessed. General and neurological examination, laboratory tests, and a brain magnetic resonance imaging resulted in normal ranges. An electroencephalogram showed slow waves including isolated spikes in the right temporal and parietal regions. After a mild improvement of symptoms, he developed a grossly disorganized behavior, with conceptual disorganization, auditory and visual hallucinations, delusion and suspiciousness, hostility, social and emotional withdrawal, somatic concerns, anxiety, and tension. Psychopathological assessment was performed by the use of Positive and Negative Syndrome Scale (PANSS). His PANSS total score was 115, while PANSS subscale scores were 29 for positive, 26 for negative, and 60 for general psychopathological symptoms. After proper informed consent, a treatment with antipsychotics and mood stabilizers (risperidone 6 mg/day, levosulpiride 72 mg/day, valproic acid 1000 mg/day) was started, leading to progressive improvement of psychopathological symptoms. During this phase his GI symptomatology remained silent, with 2–3 regular bowel movements/day and no blood or mucus emissions.

After a 6-month follow-up, a psychopharmacological maintenance with risperidone (4 mg/day) and valproic acid (1000 mg/day) ensured a partial remission of symptomatology. His PANSS total score was 71, with subscale scores of 11 for positive symptoms, 21 for negative symptoms and 39 for general psychopathological symptoms. On the other hand, only mesalazine efficiently controlled GI symptoms (2–3 regular bowel movements/day and no abdominal pain, diarrhea or rectal bleeding). Therefore, according to *Diagnostic and Statistical Manual of Mental Disorders*, Fifth Edition (DSM-5) criteria, a diagnosis of partial remission of a first episode of schizoaffective disorder was formulated.

## Discussion

Several lines of evidence suggest that a proportion of psychotic illnesses have a plausible autoimmune component [5], although its definition and role for treatment are still a matter of debate. This case report supports the idea that, although the co-existence of a schizoaffective disorder and UC may be an accidental coincidence, their co-occurrence, early onset, and high severity, are in line with growing evidence supporting the relationship between inflammation, autoimmunity, and psychosis [17]. Thus, we raise the question of whether UC is a risk factor for the schizoaffective disorders and/or whether these two different clinical conditions may be considered two distinct manifestations of a common pathway. The clinical history of our patient indicates that UC preceded the onset of psychosis by approximately 2 years, in absence of additional risk factors for schizophrenia spectrum disorders (psychotic illness in relatives, obstetric complications, abnormalities of early phases of neurodevelopment, gradual impairment in academic or psychosocial functioning). This temporal relationship, suggestive of a risk for immunologically mediated psychotic disorders, also emerges from a large Danish study [18] showing that several autoimmune diseases, including IBD, were associated with an increased risk of developing bipolar disorder or schizophrenia within 4–5 years from diagnosis. In addition, some previous data supported the hypothesis of an association between central nervous system infections in childhood and increased risk of schizophrenia related to abnormalities in immune mediators such as cytokines and chemokines or raised antibodies, triggered by a postnatal viral infection [19]. On the other hand, schizophrenia and immune disorders share some genetic factors. A polygenic risk score analysis suggested that a spectrum of genetic factors are shared by schizophrenia and different disorders characterized by immune dysregulation [20]. In fact, studies on schizophrenia revealed an intricate association of environmentally driven immune activation and genetically encoded immune dysfunctions. The intestinal mucosa is part of an intricate enteric immune system and is endowed with a large variety of immune cells [21]. As the largest immune organ in the body, the GI tract is a plausible junction to reconcile hypotheses regarding how the autoimmune response and GI-related products can become neuropathogenic. GI inflammation affects endothelial permeability and provides a means by which gut-derived products might penetrate the blood-brain barrier. These faulty barriers may be particularly important if autoantibodies generated in the inflamed gut would cross a compromised blood–brain barrier [6, 22]. Despite the growing evidence that increased intestinal permeability with subsequent immune activation has a major role in the pathophysiology of various psychiatric disorders [23], to date, the “leaky gut” hypothesis needs further demonstrations.

Moreover, considering IBD across the age spectrum, some authors proposed that, especially in younger patients, genetics might play a more significant role as compared to other components, such as the immune system, environmental factors, and composition of the intestinal microbiota [24]. On the basis of these observations, we hypothesize that the increased genetic vulnerability characterizing IBD may also increase the risk of developing psychotic diseases and that the co-occurrence of UC and schizoaffective disorder may arise from a common pathological pathway deriving from brain–gut interactions.

One last consideration must be made on the relationship between pharmacological treatment of UC and psychotic onset. After a long period of resistance to conventional therapies, a good clinical response was achieved after treatment with thalidomide, an immunomodulatory drug used in the experimental treatment of refractory IBD. At the same time, after approximately 2 months of treatment with thalidomide, our patient developed an acute onset of FEP. The timing of such events might suggest that the psychotic symptoms are adverse events after thalidomide treatment. Of note, two different systematic reviews on the efficacy and safety of thalidomide in the treatment of IBD reported that among the most frequent adverse events are neurological disturbances, such as sedation and peripheral neuropathy, without any report of psychotic symptoms [25, 26]. Therefore, on the basis of current data, it is not possible to exclude that thalidomide may have played a role in promoting psychotic manifestations in an individual with a specific vulnerability to schizoaffective disorders.

To the best of our knowledge this is the first case report of an adolescent patient presenting with early onset UC and a schizoaffective disorder. Questions arise from our report regarding whether and how immunological changes correlate with the clinical course of schizophrenia spectrum disorders, potentially modifying the current dogma about disease pathogenesis and leading to novel disease prevention and treatment strategies. Finally, we suggest that particular attention must be placed on the potential effects that immunomodulatory drugs might exert on autoimmunity, inflammation, and psychosis.

## Abbreviations

FEP: First-episode psychosis
GI: Gastrointestinal
IBD: Inflammatory bowel disease
PANSS: Positive and Negative Syndrome Scale
UC: Ulcerative colitis
